# Reconsidering the prognostic significance of tumour deposit count in the TNM staging system for colorectal cancer

**DOI:** 10.1038/s41598-019-57041-2

**Published:** 2020-01-09

**Authors:** Song Wang, Xu Guan, Mingfei Ma, Meng Zhuang, Tianyi Ma, Zheng Liu, Haipeng Chen, Zheng Jiang, Yinggang Chen, Guiyu Wang, Xishan Wang

**Affiliations:** 1 Department of Colorectal Surgery, The Second Affiliated Hospital of Harbin Medical University, Harbin, Heilongjiang 150081 China; 2 Department of Colorectal Surgery, Cancer Hospital, Chinese Academy of Medical Sciences and Peking Union Medical College, Beijing, 100021 China; 3 Medical Department, Heilongjiang University of Chinese Medicine, Harbin, Heilongjiang 150040 China

**Keywords:** Colorectal cancer, Colorectal cancer, Surgical oncology, Surgical oncology

## Abstract

Although the occurrence of tumour deposits (TDs) without metastatic lymph nodes (mLNs) is classified as “N1c” in the 8^th^ TNM staging system for colorectal cancer (CRC), the prognostic significance of the TD count is still controversial. A total of 39155 CRC patients were collected from the Surveillance, Epidemiology, and End Results (SEER) database. The potential associations between baseline characteristics and TD status were evaluated using the χ^2^ test. Cancer-specific survival (CSS) rates were calculated by using the Kaplan-Meier method, and CSS comparisons were performed by using the log-rank test. The results showed that TD count was an important prognostic factor and that the number of TDs was negatively correlated with the prognosis of CRC patients. We found that the prognostic value of one TD is equivalent to that of two mLNs based on the comparison of CSS rates. Accordingly, we proposed a novel N staging system by integrating the TD count into the N category with the ratio of TDs to mLNs being 1:2. There were no prognostic differences in patients with or without TDs in each novel N category. Weighing one TD as two mLNs in this novel TNM staging system is superior to the “N1c” classification in the 8^th^ TNM staging system in evaluating the prognosis of CRC patients.

## Introduction

The TNM staging system is an important determinant in judging the prognosis and guiding treatment of colorectal cancer (CRC) and is widely used worldwide^1^. However, over the past 20 years, the TNM staging system has changed dramatically from the 5^th^ edition to the 8^th^ edition. During this period, the main controversy is concentrated on tumour deposits (TDs), which are nodules without histologic evidence of lymph node structures^2,3^, and these occur in 20% of CRC patients who undergo radical surgery. Even the latest classification criterion of TDs is still questioned by many scholars.

In 1997, TDs were first put forward in the 5^th^ edition of the TNM staging system for CRC^4^; TDs greater than 3 mm were considered an N factor, and those not exceeding 3 mm as a T factor. In the 6^th^ edition (2002), contour characteristics of TDs were identified as a new classification criterion^5^. TDs should be classified into the N category if the nodule has a form and smooth contour of a lymph node and into the T category if the nodule has an irregular contour. However, the standpoint of dividing TDs into T and N factors based on the size or contour was abandoned, and a new category, “N1c”, was proposed since the 7^th^ edition (2009)^6^. N1c refers to TDs in the subserosa, mesentery, nonperitonealized pericolic or perirectal tissues without regional nodal metastasis. Although the latest TNM staging system (8^th^ edition) did not change the TD classification^7^, an increasing number of studies state that the “N1c” category could not perfectly reflect the role of TDs in the prognosis^8–10^. Some authors believed that the number of TDs should be recorded regardless of whether the patients had lymph node metastasis.

In light of these considerations, the aims of this study are to estimate the likelihood of counting TDs as metastatic lymph nodes (mLNs) and to reconsider the prognostic value of TD count in the TNM staging system. Finally, we propose a new staging method that can more accurately predict the prognosis of CRC patients.

## Results

A total of 39155 CRC patients were collected from the Surveillance, Epidemiology, and End Results (SEER) database, including 2863 patients with TDs and 36292 patients without TDs. There were no significant differences between the TD (+) group and the TD (−) group regarding the characteristics of sex and race. In the TD (+) group, 1819 patients (63.5%) were ages <70 years, 2668 patients (93.2%) were classified as T3/T4, 1007 patients (35.2%) were classified as N2, 776 patients (27.1%) had grade III/IV tumour, 201 patients (7.0%) had mucinous adenocarcinoma, 37 patients (1.4%) had signet ring cell carcinoma and 859 patients (30.0%) were classified as M1; the proportions of these characteristics were obviously higher than those in the counterparts of the TD (−) group. In addition, colon cancer patients accounted for 72.3% of the TD (+) group, which was a lower percentage than that of the TD (−) group. Detailed information is listed in Table 1.

**Table 1:** Characteristic comparisons between patients in the TD (+) and TD (−) groups. Note: Grade I/II are analogous to well diferentiated or moderately differentiated, and grade III/IV are analogous to poorly diferentiated or undifferentiated. Abbreviations: AJCC, American Joint Committee on Cancer system.

Characteristics	Tumour Deposit Status				P Value	
	Positive		Negative		Univariate Analysis	Multivariate Analysis
Sex					0.437	0.681
Male	1490	52.0%	18614	51.3%		
Female	1373	48.0%	17678	48.7%		
Age (Years)					0.000	0.960
<70	1819	63.5%	21217	58.5%		
≥70	1044	36.5%	15075	41.5%		
Race					0.566	0.104
Black	304	10.6%	3687	10.2%		
White	2148	75.0%	27187	74.9%		
Other	411	14.4%	5418	14.9%		
AJCC T Stage					0.000	0.000
T1/T2	195	6.8%	12207	33.6%		
T3/T4	2668	93.2%	24085	66.4%		
AJCC N Stage					0.000	0.000
N0	250	8.7%	23021	63.4%		
N1	1606	56.1%	8823	24.3%		
N2	1007	35.2%	4448	12.3%		
AJCC M Stage					0.000	0.000
M0	2004	70.0%	33012	91.0%		
M1	859	30.0%	3280	9.0%		
AJCC TNM Stage					0.000	0.000
Stage I	25	0.9%	10196	28.1%		
Stage II	186	6.5%	11996	33.1%		
Stage III	1793	62.6%	10820	29.8%		
Stage IV	859	30.0%	3280	9.0%		
Grade					0.000	0.042
Grade I/II	2087	72.9%	30139	83.0%		
Grade III/IV	776	27.1%	6153	17.0%		
Histological Type					0.000	0.970
Adenocarcinoma	2595	90.6%	33488	92.3%		
Mucinous adenocarcinoma	201	7.0%	2281	6.3%		
Signet ring cell carcinoma	37	1.3%	243	0.7%		
Other	30	1.0%	280	0.8%		
Location					0.002	0.001
Colon	2071	72.3%	27190	74.9%		
Rectum	792	27.7%	9102	25.1%		

### Survival comparisons between TD (+) and TD (−) patients

**Figure 1:**
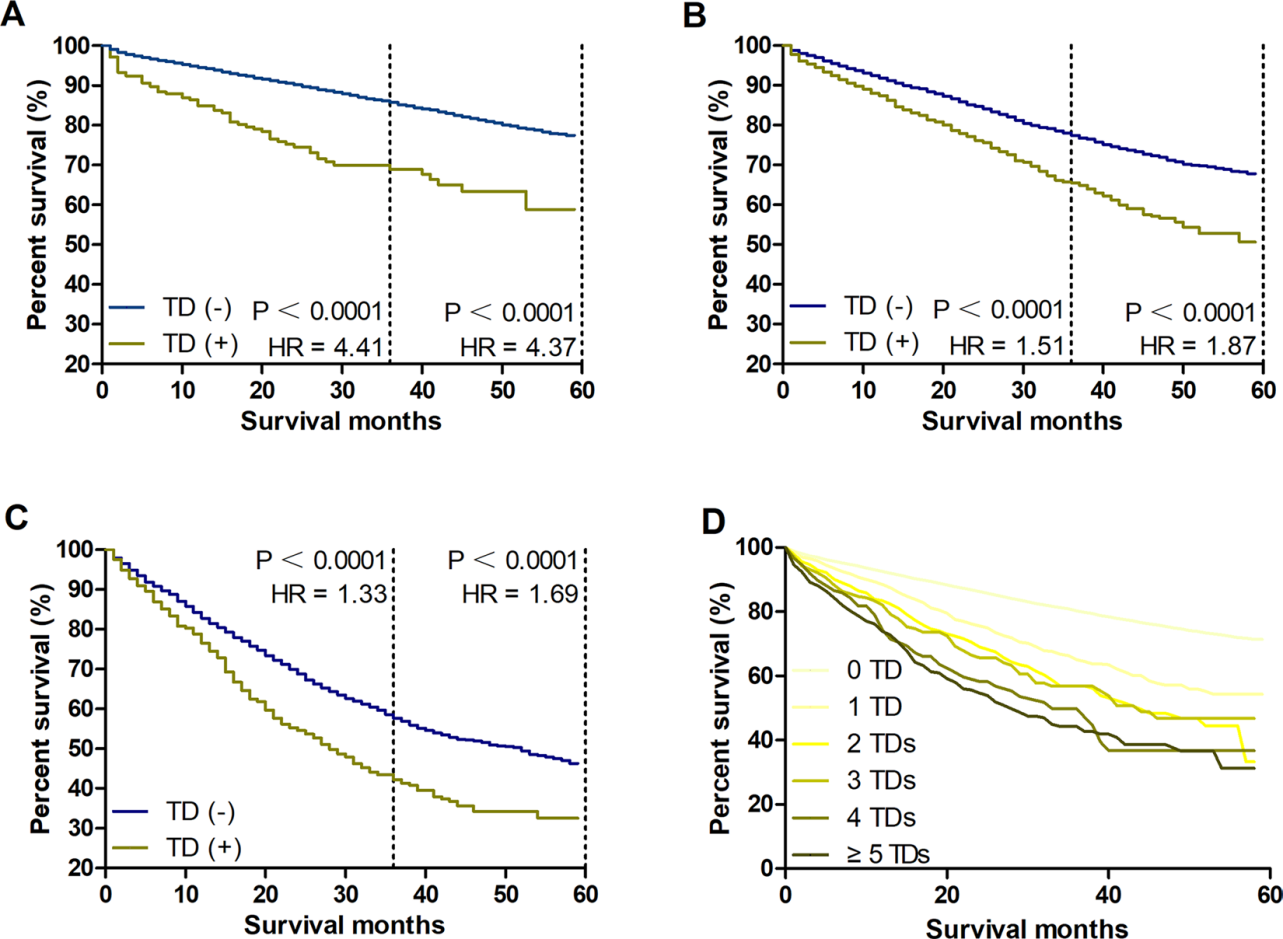
Survival comparisons between TD (+) and TD (−) patients. (**A**) Survival comparison between TD (+) and TD (−) patients without mLNs. (**B**) Survival comparison between TD (+) and TD (−) patients with 1–3 mLNs. (**C**) Survival comparison between TD (+) and TD (−) patients with ≥4 mLNs. (**D**) Survival comparison among patients with different numbers of TDs. HR: hazard ratio.

The CRC patients were divided into three subgroups: patients without mLN, patients with 1–3 mLNs, and patients with ≥4 mLNs. Then, the cancer-specific survival (CSS) was compared between TD (+) and TD (−) patients among these three subgroups. Here, the results showed that the 3- and 5-year CCS rates of TD (+) patients were obviously lower than those of TD (−) patients in the three subgroups (*P* < 0.0001) (Fig. 1A–C). Furthermore, the 5-year CSS rates were significantly decreased in those patients with increasing numbers of TDs (Fig. 1D). This result indicated that TD was not only an important prognostic factor for CRC patients, but also TD count should be fully considered in the evaluation of the TD status.

### Exploring the prognostic value of TDs

**Figure 2:**
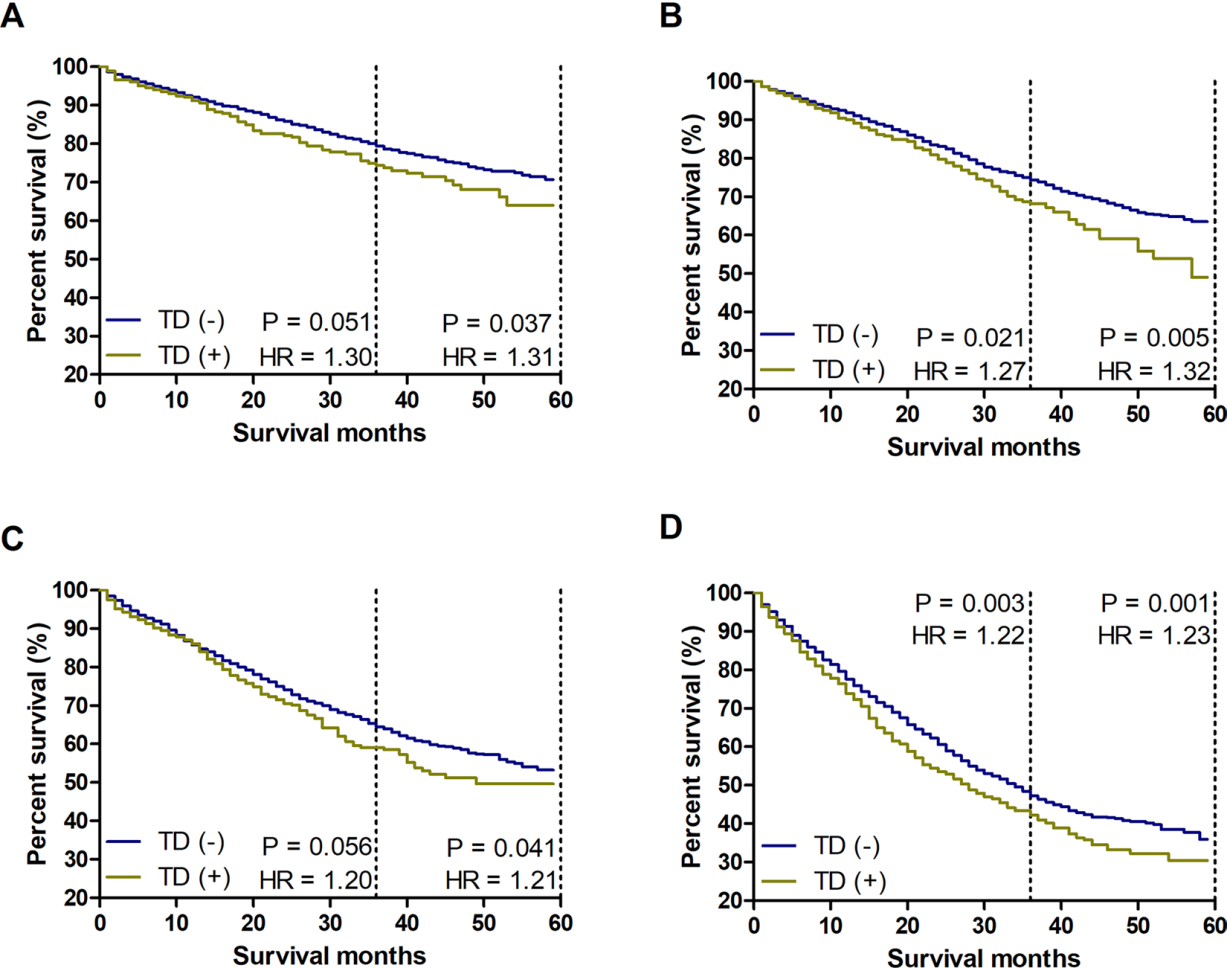
Survival comparisons between TD (+) and TD (−) patients with the same number of mLNs. The number of mLNs is equal to the sum of real mLNs and TDs. (**A**) Survival comparison between TD (+) and TD (−) patients with 1 mLN. (**B**) Survival comparison between TD (+) and TD (−) patients with 2–3 mLNs. (**C**) Survival comparison between TD (+) and TD (−) patients with 4–6 mLNs. (**D**) Survival comparison between TD (+) and TD (−) patients with ≥7 mLNs.

Due to the influence of counting TDs on the prognosis, we integrated the number of TDs into the TNM staging system to substitute the current “N1c” to further establish a more scientific and reasonable TNM staging system. However, it is essential to explore the prognostic correlation between the number of mLNs and the number of TDs. First, we supposed that the prognostic value of one TD was equivalent to that of one mLN. To examine whether one TD possesses the same weight as one mLN in terms of prognosis, we compared the CSS between patients with TDs and patients without TDs in the new N category. Here, the total number of mLNs was equal to the sum of real mLNs and TDs. The results showed that the CSS of TD (+) patients was significantly worse than that of TD (−) patients (Fig. 2), which indicated that the ratio of 1:1 for mLNs to TDs did not accurately define the prognostic value of TDs for CRC patients.

**Figure 3:**
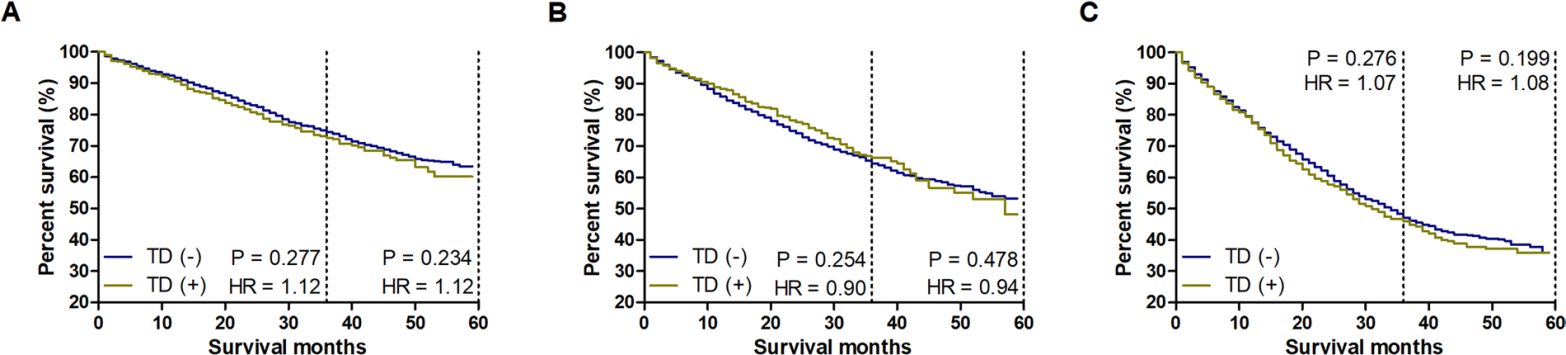
Survival comparisons between TD (+) and TD (−) patients with the same number of mLNs. The number of mLNs is equal to the sum of the number of real mLNs and double the number of TDs. (**A**) Survival comparison between TD (+) and TD (−) patients with 2–3 mLNs. (**B**) Survival comparison between TD (+) and TD (−) patients with 4–6 mLNs. (**C**) Survival comparison between TD (+) and TD (−) patients with ≥7 mLNs.

To further explore the prognostic weight of TDs, we supposed again that the prognostic value of one TD was equal to that of two mLNs. Then, we compared the CSS between patients with TDs and patients without TDs in each novel N category. Here, the results showed that the CSS rates of patients with TDs and without TDs were consistent among these subgroups (Fig. 3). This suggested that the prognostic value of one TD was equivalent to that of two mLNs.

### Validating the prognostic value of TDs

**Figure 4:**
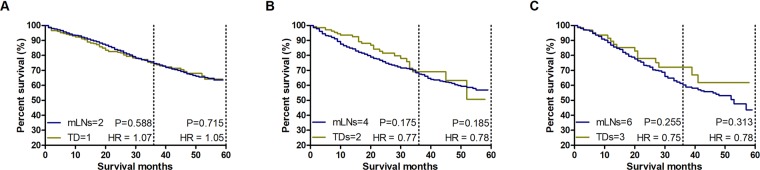
Validating the prognostic value of TDs. (**A**) Survival comparison between patients with only 1 TD and patients with only 2 mLNs. (**B**) Survival comparison between patients with only 2 TDs and patients with only 4 mLNs. (**C**) Survival comparison between patients with only 3 TDs and patients with only 6 mLNs.

To further verify the prognostic value of TDs, we directly and separately compared the CSS rates between patients with only 1 TD and patients with only 2 mLNs, between patients with only 2 TDs and patients with only 4 mLNs, between patients with only 3 TDs and patients with only 6 mLNs. We found no survival differences between TDs and mLNs among these compared groups (Fig. 4).

Although the prognostic value of one TD being equivalent to two mLNs has been determined, weighing one TD as three or four mLNs was still performed to draw a conclusion more cautiously. Supplementary Fig. S1 shows that the CSS rates of patients with TDs were consistent with those of patients without TDs. Nevertheless, Supplementary Fig. S2 shows that the prognosis of 2 TDs was superior to that of 6 mLNs and the prognosis of 3 TDs was superior to that of 9 mLNs. Regarding the consideration of one TD as four mLNs, the CSS of the TD (+) group was significantly higher than that of the TD (−) group (Supplementary Figs. S3, S4). Therefore, the role of TDs on prognosis was overestimated by weighing one TD as three or four mLNs. Weighing one TD as two mLNs is the best ratio.

**Figure 5:**
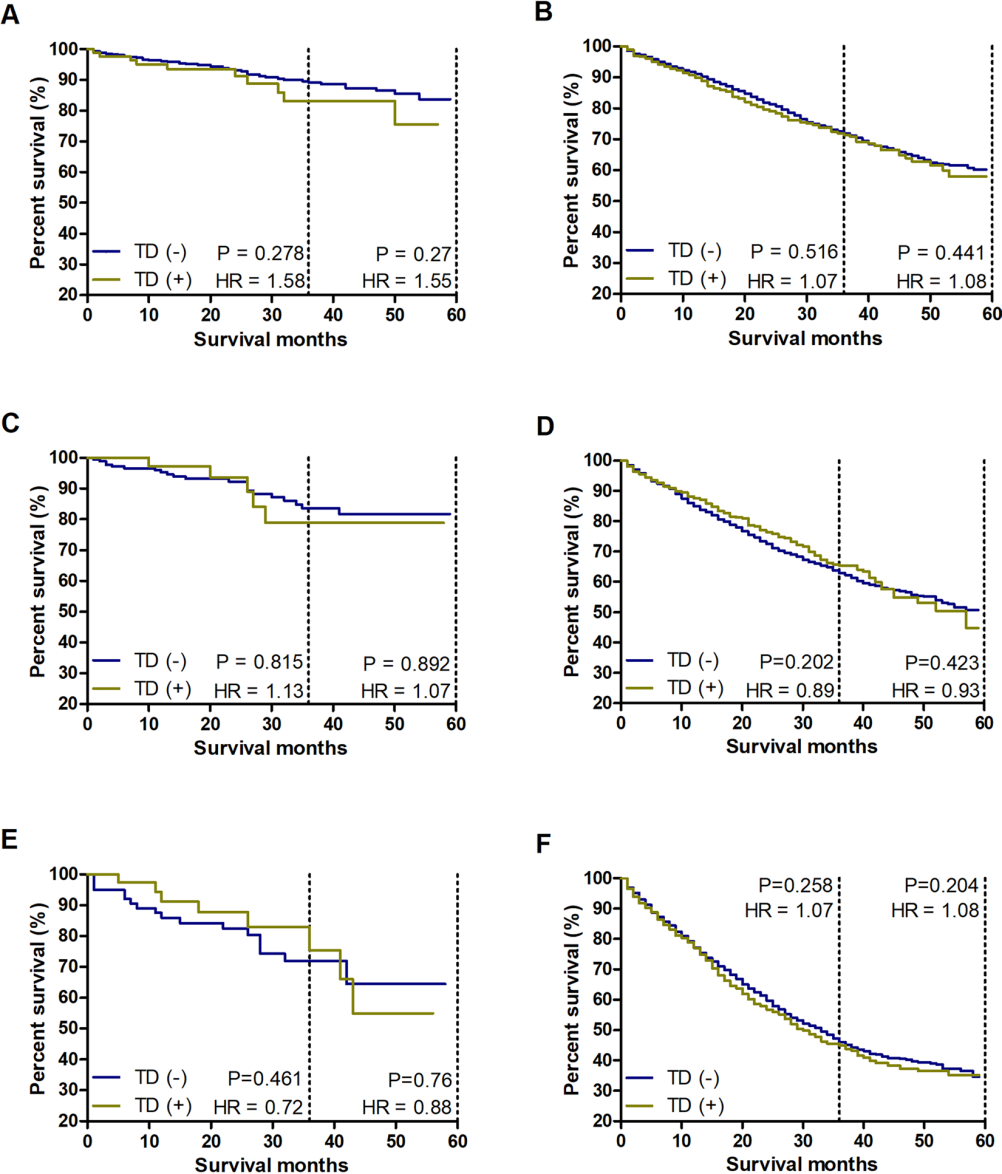
Survival comparison between TD (+) and TD (−) patients in every T category with the same amount of mLNs. The number of mLNs is equal to the sum of the number of real mLNs and double the number of TDs. (**A**) Survival comparison between TD (+) and TD (−) T1/T2 patients with 2–3 mLNs. (**B**) Survival comparison between TD (+) and TD (−) T3/T4 patients with 2–3 mLNs. (**C**) Survival comparison between TD (+) and TD (−) T1/T2 patients with 4–6 mLNs. (**D**) Survival comparison between TD (+) and TD (−) T3/T4 patients with 4–6 mLNs. (**E**) Survival comparison between TD (+) and TD (−) T1/T2 patients with ≥7 mLNs. (**F**) Survival comparison between TD (+) and TD (−) T3/T4 patients with ≥7 mLNs.

To exclude the influence of T stage on N stage, survival comparisons were separately performed in every T category. The results showed that the CSS rates of patients with TDs were equal to those without TDs among these six subgroups, which further confirmed that the prognostic value of one TD being equal to that of two mLNs was reasonable and scientific (Fig. 5).

To exclude the influence of tumour location on TDs, survival comparisons were separately performed for colon cancer and rectal cancer. In the traditional N staging method of colon cancer, there was a significant difference between the TD (+) and TD (−) groups (*P* < 0.0001) (Supplementary Fig. S5). However, integrating the TD count into the N category with the ratio of TDs to mLNs of 1:2 eliminated the prognostic difference between the two groups (Supplementary Fig. S6). The same result was obtained for rectal cancer (Supplementary Figs. S7, S8). Therefore, it is feasible and necessary to regard a TD as two mLNs in both colon and rectal cancers.

### The establishment of a novel N staging system

**Figure 6:**
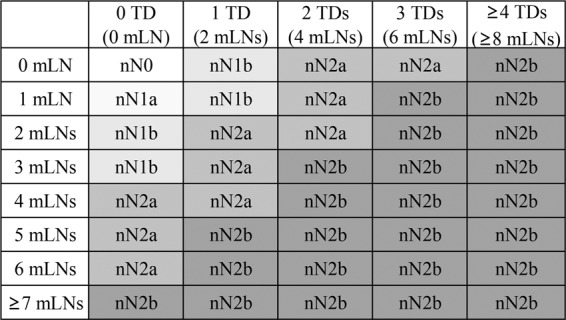
The novel N staging system.

Due to the prognostic value of 1 TD being equivalent to that of 2 mLNs, the number of TDs was integrated into the N stage with a ratio of TDs to mLNs of 1:2. Therefore, the novel N stage was established as follows: nN0 (no mLNs and no TDs), nN1a (1 mLN), nN1b (the sum of mLNs and double the number of TDs is equal to 2 or 3), nN2a (the sum of mLNs and double the number of TDs is equal to 4–6), and nN2b (the sum of mLNs and double the number of TDs is greater than 6); the details are shown in Fig. 6.

## Discussion

TDs were first described as an entity and considered to be the result of lymphatic tumour dissemination by Gabriel *et al*. in 1935^11^. Since then, TDs in adjacent adipose tissues have become a well-known feature of CRC, and many studies have investigated their origin, distribution and clinical significance^12–14^. Although scholars have performed many studies on TDs, our understanding of TDs remains limited. Some consider they are derived from growth within or along vascula, nerves or lymphatic channels of cancer cells^15^. Some believe that TDs are potentially positive lymph nodes that are no longer recognizable because of complete replacement by metastatic tumours^16^. Currently, there are many other theories concerning the origin of TDs.

Although their origin remains unclear, it is clear that TDs have been associated with poor prognosis of patients with CRC. A review of colorectal TDs in the mesorectum and pericolon of 3714 patients from 12 papers found that their incidence varied from 5% to 45%, and their status not only decreased disease-free survival (DFS) and overall survival (OS) but also increased local recurrence rates and distant metastasis rates^15^. In addition, they suggested that the presence of TDs after neoadjuvant therapy indicated a good response to the corresponding treatment. Reviewing cases of 1027 patients who had undergone curative surgery for CRC and calculating the HR (4.5 for TDs) through Cox proportional regression analysis, Hideki Ueno *et al*.^17^ considered that TDs played an independent prognostic role. In the present study, the CCS rates of TD (+) patients were obviously lower than those of with TD (−) patients. Consistent with previous studies, TD is associated with adverse prognosis in CRC patients.

To date, the UICC/AJCC TNM staging system has proposed several categorization criteria for TDs, such as classifying TDs into T and N factors by their size or contours. However, clinical studies have reported that the presence of a TD had little influence and clinical significance on the T stage. A multicentre retrospective study performed for 1716 CRC patients from eleven institutions as the first cohort and an additional 2242 patients from nine institutions as the second cohort demonstrated that neither the 3 mm rule nor the contour rule provided convincing advantages in tumour staging^18^. We believe that it is unreasonable to treat any type of TD as a T category. Furthermore, classifying TDs as mLNs into N category will aid CRC prognosis and treatment.

There were significant differences in prognosis between patients with and without TDs in this study (P < 0.0001), regardless of which group (no mLNs, 1–3 mLNs, or ≥4 mLNs) they belonged to. Therefore, the incorporation of TDs into the N category should be accepted. The latest TNM staging system defined the presence of TDs as N1c in patients without mLNs. However, this system neglected the effect of TDs on the adverse outcomes of patients with mLNs^19^. Goldstein and Turner^20^ studied 418 T3N + M0 colon adenocarcinomas, containing 71 (18%) patients with TDs. The results showed that the 5-year DFS of patients with no TDs, 1 or 2 TDs, and 3 or more TDs was 35%, 24%, and 2%, respectively (P < 0.01). Both the presence and the number of TDs were associated with recurrence, metastasis and survival of CRC patients. Consistent with our results, the greater the number of TDs, the worse the prognosis of patients. One group of authors proposed a novel N category in which TDs were counted as mLNs based on a retrospective study of 513 patients with CRC^21^. Their novel N category could reduce prognostic differences between the two groups but could not eliminate them.

Currently, most studies concerning TDs are based on small sample sizes or in a single institution. We proposed an optimal staging method according to SEER, a database with large datasets from multiple institutions, to predict prognosis and guide treatment more accurately. The results suggested that the prognostic value of a TD was equivalent to that of 2 mLNs. We put forward a novel TNM staging system, counting each TD as 2 mLNs, which is superior to any previous staging method. Nevertheless, our study emphasizes the high weight of TDs compared with mLNs on the poor outcome of advanced CRC and provides data to update the UICC/AJCC guidelines. Before being widely used, further studies are needed to investigate this staging method.

## Conclusion

From this study, we can draw a conclusion that weighing one TD as two mLNs in the novel TNM staging system is superior to the “N1c” classification in the 8^th^ TNM staging system in evaluating the prognosis of CRC patients.

## Methods

### Study population

We extracted the cancer cases from the SEER database. The SEER database includes the incidence, demographic, therapy and survival data from 17 cancer registries in the United States. We have obtained permission to obtain a research data file in the SEER database from the National Cancer Institute, and the reference number is 11738-Nov2016. The design of this study was approved by the Ethics Committees of the included hospitals.

In this study, CRC patients in stages I to IV were identified from January 2010 to December 2014 because the TD status was available in the SEER database since 2010. All CRC patients collected in this study received radical surgery to confirm the accuracy of a pathological evaluation. The TD status was categorized dichotomously as ‘negative’ or ‘positive’. Negative TD status indicates that no TDs were found, and positive TD status represents that at least one TD was identified. The other information extracted from the SEER database included general information (sex, age, race and year of diagnosis), tumour pathology information (depth of tumour invasion, number of positive regional nodes, distant metastasis, tumour location, tumour grade and tumour histology), and survival data (CSS, which is defined as the period from cancer diagnosis until death due to a cancer cause). Excluded from this study included patients who received preoperative chemoradiotherapy to avoid the decreased number of lymph nodes and TDs, patients with unknown TD status and patients who died due to other causes.

### Statistical analysis

The potential associations between baseline characteristics and TD status were evaluated using the χ^2^ test. Multivariate analysis was performed using a binomial logistic regression model. CSS rates were calculated by using the Kaplan-Meier method, and CSS comparisons were performed by using the log-rank test. The SPSS statistical software package (version 20.0) was used for all statistical analyses. A P-value less than 0.05 was considered statistically significant.

### Ethics approval and consent to participate

This study was approved by the Institutional Review Board of National Cancer Center/Cancer Hospital, Chinese Academy of Medical Sciences and Peking Union Medical College and the Second Affiliated Hospital of Harbin Medical University. All methods were carried out in accordance with the Declaration of Helsinki and the approved guidelines. The data enrolled in this study were obtained from the openly accessed SEER database, and the National Cancer Institute gave us permission to obtain data files for research only (Reference number: 11738-Nov2016). Ethical committees determined that this article was not a human participant research study and did not include personal identifying information. Therefore, this study does not require informed consent.

## Supplementary information

Supplementary information.

## Data Availability

Data are available in the Surveillance, Epidemiology, and End Results cancer registry (https://seer.cancer.gov) and can also be provided by the corresponding authors.
