# Supplementary material for: Reconsidering the prognostic significance of tumour deposit count in the TNM staging system for colorectal cancer
Source: Sci Rep. 2020 Jan 9;10:89. doi: 10.1038/s41598-019-57041-2 (PMC6952424; doi:10.1038/s41598-019-57041-2)
Supplement: Supplementary file 1 — Supplementary information. [file 41598_2019_57041_MOESM1_ESM.pdf]

Reconsidering the prognostic significance of tumour deposit count in the TNM staging system for colorectal cancer

Song Wang[1*], Xu Guan[2*], Mingfei Ma[3], Meng Zhuang[2], Tianyi Ma[1], Zheng Liu[2], Haipeng Chen[2], Zheng Jiang[2], Yinggang Chen[1], Guiyu Wang[1], Xishan Wang[1,2]

[1]Department of Colorectal Surgery, the Second Affiliated Hospital of Harbin Medical University, Harbin, Heilongjiang 150081, China. [2]Department of Colorectal Surgery, Cancer Hospital, Chinese Academy of Medical Sciences and Peking Union Medical College, Beijing 100021, China. [3]Medical Department, Heilongjiang University of Chinese Medicine, Harbin, Heilongjiang 150040, China. [*]These authors contributed equally to this manuscript. Correspondence and requests for materials should be addressed to X.W. (Email: wxshan1208@126.com), G.W. (Email: guiywang@163.com) or H.C. (Email: 214787653@qq.com)

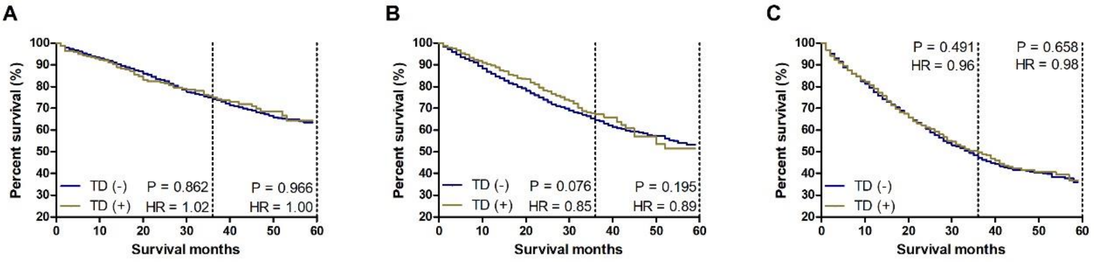

Supplementary Figure 1. Survival comparisons between TD (+) and TD (-) patients with the same number of mLNs. The number of mLNs is equal to the sum of the number of real mLNs and triple the number of TDs. (**A**) Survival comparison between TD (+) and TD (-) patients with 2-3 mLNs. (**B**) Survival comparison between TD (+) and TD (-) patients with 4-6 mLNs. (**C**) Survival comparison between TD (+) and TD (-) patients with ≥7 mLNs.

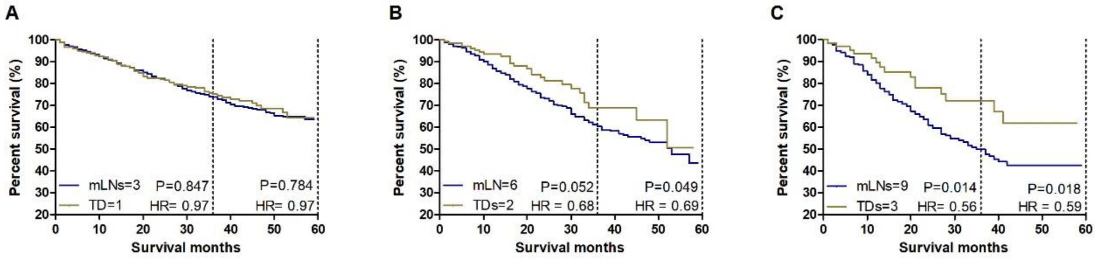

Supplementary Figure 2. Validating the prognostic value of TDs. (**A**) Survival comparison between patients with only 1 TD and patients with only 3 mLNs. (**B**) Survival comparison between patients with only 2 TDs and patients with only 6 mLNs. (**C**) Survival comparison between patients with only 3 TDs and patients with only 9 mLNs.

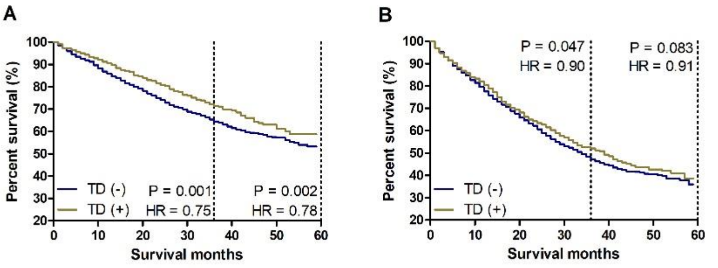

Supplementary Figure 3. Survival comparisons between TD (+) and TD (-) patients with the same number of mLNs. The number of mLNs is equal to the sum of the number of real mLNs and four times the number of TDs. (**A**) Survival comparison between TD (+) and TD (-) patients with 4-6 mLNs. (**B**) Survival comparison between TD (+) and TD (-) patients with ≥7 mLNs.

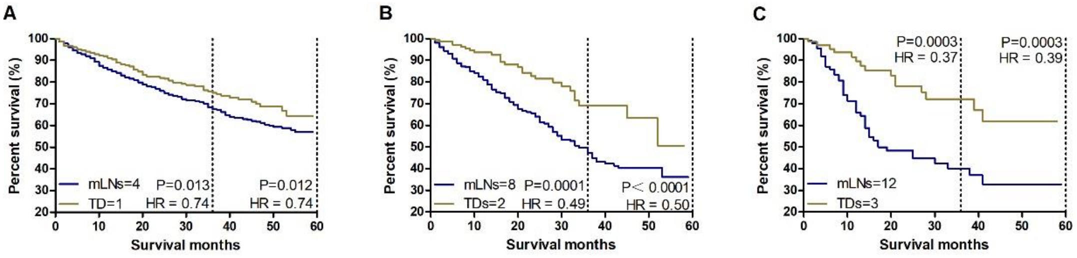

Supplementary Figure 4. Validating the prognostic value of TDs. (**A**) Survival comparison between patients with only 1 TD and patients with only 4 mLNs. (**B**) Survival comparison between patients with only 2 TDs and patients with only 8 mLNs. (**C**) Survival comparison between patients with only 3 TDs and patients with only 12 mLNs.

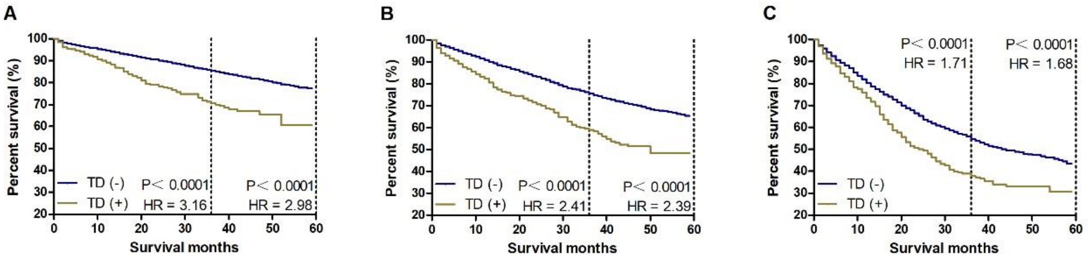

Supplementary Figure 5. Survival comparisons between TD (+) and TD (-) patients with colon cancer. (**A**) Survival comparison between TD (+) and TD (-) in colon cancer patients without mLNs. (**B**) Survival comparison between TD (+) and TD (-) in colon cancer patients with 1-3 mLNs. (**C**) Survival comparison between TD (+) and TD (-) in colon cancer patients with ≥4 mLNs.

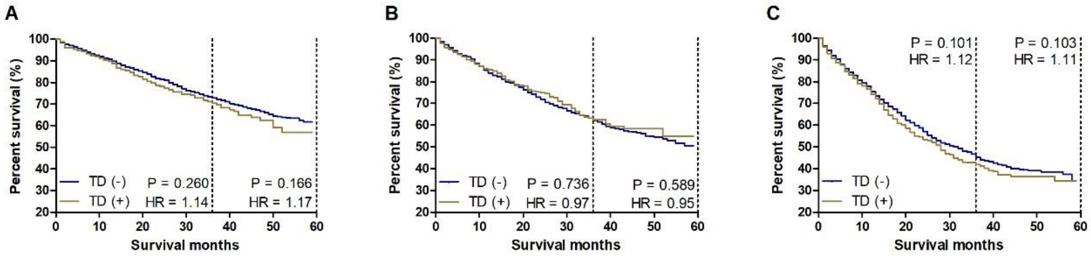

Supplementary Figure 6. Survival comparisons between TD (+) and TD (-) in colon cancer patients with the same number of mLNs. The number of mLNs is equal to the sum of the number of real mLNs and double the number of TDs. (**A**) Survival comparison between TD (+) and TD (-) in colon cancer patients with 2-3 mLNs. (**B**) Survival comparison between TD (+) and TD (-) in colon cancer patients with 4-6 mLNs. (**C**) Survival comparison between TD (+) and TD (-) in colon cancer patients with ≥7 mLNs.

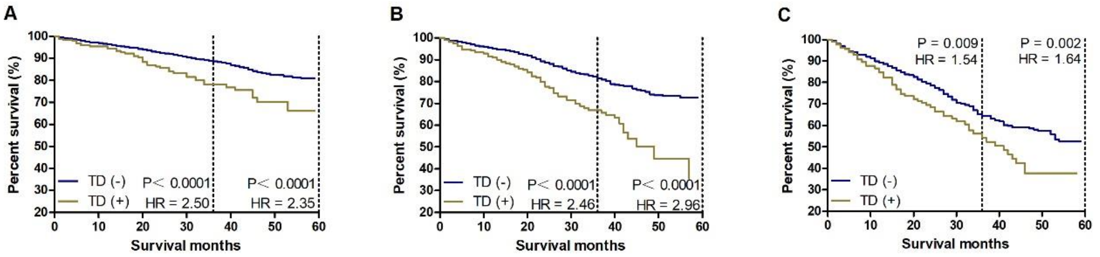

Supplementary Figure 7. Survival comparisons between TD (+) and TD (-) patients with rectal cancer. (**A**) Survival comparison between TD (+) and TD (-) in rectal cancer patients without mLNs. (**B**) Survival comparison between TD (+) and TD (-) in rectal cancer patients with 1-3 mLNs. (**C**) Survival comparison between TD (+) and TD (-) in rectal cancer patients with ≥4 mLNs.

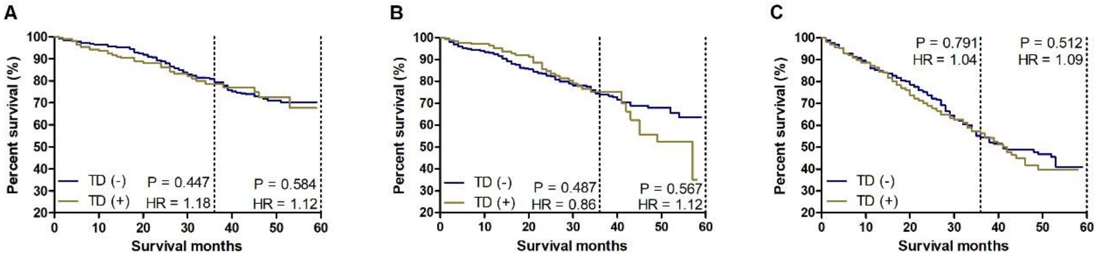

Supplementary Figure 8. Survival comparisons between TD (+) and TD (-) in rectal cancer patients with the same number of mLNs. The number of mLNs is equal to the sum of the number of real mLNs and double the number of TDs. (**A**) Survival comparison between TD (+) and TD (-) in rectal cancer patients with 2-3 mLNs. (**B**) Survival comparison between TD (+) and TD (-) in rectal cancer patients with 4-6 mLNs. (**C**) Survival comparison between TD (+) and TD (-) in rectal cancer patients with ≥7 mLNs.
